# Global cancer burden growing, amidst mounting need for services

**Published:** 2024-03

**1 February 2024** - Ahead of World Cancer Day, the World Health Organization (WHO)’s cancer agency, the International Agency for Research on Cancer (IARC), released the latest estimates of the global burden of cancer. WHO also published survey results from 115 countries, showing a majority of countries do not adequately finance priority cancer and palliative care services, as part of universal health coverage (UHC).

The IARC estimates, based on the best sources of data available in countries in 2022, highlight the growing burden of cancer, the disproportionate impact on underserved populations, and the urgent need to address cancer inequities worldwide.

In 2022, there were an estimated 20 million new cancer cases and 9.7 million deaths. The estimated number of people who were alive within 5 years following a cancer diagnosis was 53.5 million. About 1 in 5 people develop cancer in their lifetime, approximately 1 in 9 men and 1 in 12 women die from the disease.

The global WHO survey on UHC and cancer shows that only 39% of participating countries covered the basics of cancer management as part of their financed core health services for all citizens, ‘health benefit packages’ (HBP). Only 28% of participating countries additionally covered care for people who require palliative care, including pain relief in general, and not just linked to cancer.

Three major cancer types in 2022: lung, breast and colorectal cancers

The new estimates available on IARC’s Global Cancer Observatory show that 10 types of cancer collectively comprised around two-thirds of new cases and deaths globally in 2022. Data covers 185 countries and 36 cancers.

Lung cancer was the most commonly occurring cancer worldwide with 2.5 million new cases accounting for 12.4% of the total new cases. Female breast cancer ranked second (2.3 million cases, 11.6%), followed by colorectal cancer (1.9 million cases, 9.6%), prostate cancer (1.5 million cases, 7.3%), and stomach cancer (970 000 cases, 4.9%).

Lung cancer was the leading cause of cancer death (1.8 million deaths, 18.7% of the total cancer deaths) followed by colorectal cancer (900 000 deaths, 9.3%), liver cancer (760 000 deaths, 7.8%), breast cancer (670 000 deaths, 6.9%) and stomach cancer (660 000 deaths, 6.8%). Lung cancer’s re-emergence as the most common cancer is likely related to persistent tobacco use in Asia.

There were some differences by sex in incidence and mortality from the global total for both sexes. For women, the most commonly diagnosed cancer and leading cause of cancer death was breast cancer, whereas it was lung cancer for men. Breast cancer was the most common cancer in women in the vast majority of countries (157 of 185).

For men, prostate and colorectal cancers were the second and third most commonly occurring cancers, while liver and colorectal cancers were the second and third most common causes of cancer death. For women, lung and colorectal cancer were second and third for both the number of new cases and of deaths.

Cervical cancer was the eighth most commonly occurring cancer globally and the ninth leading cause of cancer death, accounting for 661 044 new cases and 348 186 deaths. It is the most common cancer in women in 25 countries, many of which are in sub-Saharan Africa. Even while recognizing varying incidence levels, cervical cancer can be eliminated as a public health problem, through the scale-up of the WHO Cervical Cancer Elimination Initiative.

Striking cancer inequity by Human Development Index (HDI)

Global estimates reveal striking inequities in the cancer burden according to human development. This is particularly true for breast cancer. In countries with a very high HDI, 1 in 12 women will be diagnosed with breast cancer in their lifetime and 1 in 71 women die of it. By contrast, in countries with a low HDI; while only one in 27 women is diagnosed with breast cancer in their lifetime, one in 48 women will die from it.

“Women in lower HDI countries are 50% less likely to be diagnosed with breast cancer than women in high HDI countries, yet they are at a much higher risk of dying of the disease due to late diagnosis and inadequate access to quality treatment,” explains Dr Isabelle Soerjomataram, Deputy Head of the Cancer Surveillance Branch at IARC.

WHO’s global survey of HBPs also revealed significant global inequities in cancer services. Lung cancer-related services were reportedly 4–7 times more likely to be included in a HBP in a high-income than a lower-income country. On average, there was a four-fold greater likelihood of radiation services being covered in a HBP of a high-income than a lower-income country. The widest disparity for any service was stem-cell transplantation, which was 12 times more likely to be included in a HBP of a high-income than a lower-income country.

“WHO’s new global survey sheds light on major inequalities and lack of financial protection for cancer around the world, with populations, especially in lower income countries, unable to access the basics of cancer care,” said Dr Bente Mikkelsen, Director of the Department of Noncommunicable Diseases at WHO. “WHO, including through its cancer initiatives, is working intensively with more than 75 governments to develop, finance and implement policies to promote cancer care for all. To expand on this work, major investments are urgently needed to address global inequities in cancer outcomes.”

Projected cancer burden increase in 2050

Over 35 million new cancer cases are predicted in 2050, a 77% increase from the estimated 20 million cases in 2022. The rapidly growing global cancer burden reflects both population ageing and growth, as well as changes to people’s exposure to risk factors, several of which are associated with socioeconomic development. Tobacco, alcohol and obesity are key factors behind the increasing incidence of cancer, with air pollution still a key driver of environmental risk factors.

In terms of the absolute burden, high HDI countries are expected to experience the greatest absolute increase in incidence, with an additional 4.8 million new cases predicted in 2050 compared with 2022 estimates. Yet the proportional increase in incidence is most striking in low HDI countries (142% increase) and in medium HDI countries (99%). Likewise, cancer mortality in these countries is projected to almost double in 2050.

“The impact of this increase will not be felt evenly across countries of different HDI levels. Those who have the fewest resources to manage their cancer burdens will bear the brunt of the global cancer burden,” says Dr Freddie Bray, Head of the Cancer Surveillance Branch at IARC.

“Despite the progress that has been made in the early detection of cancers and the treatment and care of cancer patients–significant disparities in cancer treatment outcomes exist not only between high and low-income regions of the world, but also within countries. Where someone lives should not determine whether they live. Tools exist to enable governments to prioritise cancer care, and to ensure that everyone has access to affordable, quality services. This is not just a resource issue but a matter of political will,” says Dr Cary Adams, head of UICC - Union for International Cancer Control.

Available from: https://www.who.int/news/item/01-02-2024-global-cancer-burden-growing--amidst-mounting-need-for-services

